# LC-HRMS based approach to identify novel sphingolipid biomarkers in breast cancer patients

**DOI:** 10.1038/s41598-020-61283-w

**Published:** 2020-03-13

**Authors:** Priyanka Bhadwal, Divya Dahiya, Dhananjay Shinde, Kim Vaiphei, Raviswamy G. H. Math, Vinay Randhawa, Navneet Agnihotri

**Affiliations:** 1 Department of Biochemistry, Panjab University, Chandigarh, India; 2 Department of General Surgery, PGIMER, Chandigarh, India; 3 Department of Pathology and Microbiology, University of Nebraska Medical Centre, Omaha, USA; 4 Department of Histopathology, PGIMER, Chandigarh, India; 5 Mass Spectrometry Facility, NCBS/InStem, Bangalore, India

**Keywords:** Biochemistry, Cancer

## Abstract

Perturbations in lipid metabolic pathways to meet the bioenergetic and biosynthetic requirements is a principal characteristic of cancer cells. Sphingolipids (SPLs) are the largest class of bioactive lipids associated to various aspects of tumorigenesis and have been extensively studied in cancer cell lines and experimental models. The clinical relevance of SPLs in human malignancies however is still poorly understood and needs further investigation. In the present study, we adopted a UHPLC-High resolution (orbitrap) Mass spectrometry (HRMS) approach to identify various sphingolipid species in breast cancer patients. A total of 49 SPLs falling into 6 subcategories have been identified. Further, integrating the multivariate analysis with metabolomics enabled us to identify an elevation in the levels of ceramide phosphates and sphingosine phosphates in tumor tissues as compared to adjacent normal tissues. The expression of genes involved in the synthesis of reported metabolites was also determined in local as well as TCGA cohort. A significant upregulation in the expression of CERK and SPHK1 was observed in tumor tissues in local and TCGA cohort. Sphingomyelin levels were found to be high in adjacent normal tissues. Consistent with the above findings, expression of SGMS1 in tumor tissues was downregulated in TCGA cohort only. Clinical correlations of the selected metabolites and their performance as biomarkers was also evaluated. Significant ROC and positive correlation with Ki67 index highlight the diagnostic potential and clinical relevance of ceramide phosphates in breast cancer.

## Introduction

Dysregulation of lipid homeostasis has become an established hallmark of cancer. The cancer cells exploit lipid metabolic pathways in order to fulfil their demand for energy as well as biosynthetic precursors. Aberrations in lipid metabolism thus affects numerous cellular processes such as cell growth, proliferation, differentiation and cell survival^1^. Sphingolipids (SPLs), apart from the inceptive view of being considered as mere structural components, have evolved as crucial regulators of myriads of cellular functions. The primary elements of sphingolipid metabolism such as ceramide, ceramide 1-phosphate and sphingosine 1-phosphate have recently emerged as key regulators in cancer cell growth, proliferation, survival, migration and drug resistance^2–4^. Numerous studies have described the elementary role of ceramide and sphingosine 1-phosphate rheostat in various types of cancers such as lung, breast and colon. However, these studies have provided the mechanistic details of sphingolipid metabolism in cancer cell lines and experimental models^5–7^, while their role in human malignancies is still poorly understood and needs further elucidation.

Breast cancer continues to remain the most common malignancy among women worldwide^8^. The diagnosis rate of breast cancer among Indian females is as high as 25.8 per 100,000 women with a mortality rate of 12.7 per 100,000 women^9^. Despite advancement in diagnostic and therapeutic modalities, the incidence of breast cancer is still expanding at an alarming rate. Therefore, the scientific quest continues for the discovery of biomarkers that can be useful in diagnosis or prediction of the disease with optimal sensitivity and specificity. As the role of SPLs has been extensively studied in various cancers, their metabolites can be envisioned as potential biomarkers in breast cancer. Recent advances in lipidomics have unraveled a reliable and informative method for the comprehensive profiling of SPLs. However, the main drawback of the technique is that it generates highly complex and large volume of the data which further requires coupling to analytical strategies to put the data into context. Though an elevation in the levels of bioactive SPLs has been reported earlier in human breast cancer patient samples^10^, a little is known about the use of these metabolites as clinically relevant biomarkers. In the previous study, the authors have used a targeted and quantitative approach to understand the difference in sphingolipid profiling between tumor and adjacent normal tissues. Our study, on the other hand, has used a non-targeted method using Ultra-High-Performance Liquid Chromatography (UHPLC)-High Resolution (orbitrap) Mass Spectrometry (HRMS) for the comprehensive profiling of SPLs in breast cancer patient samples. In addition, we have utilized more appropriate statistical tools which is a prerequisite for the biomarker identification^11^. The lipidomic studies in combination with multivariate analysis helped us uncover the biomarkers of clinical relevance in a highly efficient manner. The partial least square discriminant analysis (PLS-DA) method was employed to identify the tumor related alterations in sphingolipid metabolites. The performance of the selected metabolites as biomarkers was further assessed using receiver operating characteristics (ROC) analysis. The levels of individual SPLs were also determined among the two groups and were correlated to clinicopathological characteristics. In addition, to validate the role of selected sphingolipid metabolites in breast cancer, we also analyzed the expression of genes involved in their synthesis in the local patient cohort as well as TCGA cohort.

## Results

### Patient characteristics

A total of 31 female breast cancer patients were enrolled in the study with a median age of 50 (32–76) years. The breast cancer cohort had a greater frequency (90.3%) of invasive ductal carcinoma than those with the ductal carcinoma *in situ* (3.2%) and metastatic carcinoma (6.5%). Further, the tumor subtype grading showed that 20 (64.5%) patients were positive and 11 (35.5%) were negative for estrogen (ER) receptor. The lymph node metastasis was observed in 15 (48.4%) patients, while 16 (51.6%) patients were found to be nodal negative. The proliferation marker Ki67 was reported to be higher than 30% in 6 (19.4%) patients and less than or equal to 30% in 25 (80.6%) breast cancer patients. Overall, out of 31 patients, 21 (67.7%) were in the early (I + II) pTNM stage and 10 (32.3%) were found to be in the late stages (III + IV) of cancer (Table 1).

**Table 1:** Clinicopathological characteristics of patients with breast cancer.

**Characteristics**	
Number of patients	31
**Age (Years)**	
Median (Range)	50 (32–76)
≤50	16
>50	15
**Tumor Type**	
Ductal carcinoma *in situ* (DCIS)	1
Invasive ductal carcinoma	28
Metastatic carcinoma	2
**Tumor subtypes**	
ER+/PR+	20
ER+/PR−	6
Her2neu+	4
Triple Negative	1
**Nodal Status**	
Positive	15
Negative	16
**Ki67 (%)**	
≤30 (%)	25
>30 (%)	6
**TNM Staging**	
IA	8
IIA	10
IIB	3
IIIA	5
IIIC	3
IV	2

### Identification of sphingolipids in tumor and adjacent normal breast tissue

Comprehensive profiling of SPLs was carried out in breast tumor and adjacent normal tissues in triplicates. Based on accurate mass of precursor and fragment ions and the characteristic ions obtained, a total of 92 sphingolipid species covering 6 subcategories were identified. The species number was further decreased to 49 when the noise level was reduced to 5% and mass accuracy was set to 10 ppm for parent ion and 0.1 Da for product ions.

The characteristic fragmentation pattern of each species is summarized in Tables 2, 3 and Supplementary Tables 1 and 2.

**Table 2:** Identification of sphingolipids in breast tissue using UHPLC in “Single Phase Extract”.

Class	FattyAcid	Retention Time(min)	Molecular Formula	Observed Mass	Error (ppm)	Calculated Mass	MS/MS fragments (m/z)
**CerP**	(d23:0)	6.4	C23 H48 O6 N1 P1	464.3151	−2.15	465.3219	310,292,264
	(d23:1)	6.0	C23 H46 O6 N1 P1	462.2993	−1.73	463.3063	244, 219
	(d24:1)	6.5	C24 H48 O6 N1 P1	522.3201	−0.77	477.3219	270,122,112
	(d18:1/12:0) **(IS)**	7.0	C30 H60 O6 N1 P1	562.4229	1.24	561.4158	264,446,464
**So**	(d17:0) **(IS)**	4.5	C17 H37 O2 N1	288.2894	2.77	287.2824	270,252,240,220, 60
	(d17:1) **(IS)**	4.2	C17 H35 O2 N1	286.2737	3.14	285.2668	269,171,131,105
**S1P**	(d17:0) **(IS)**	5.2	C17 H38 O5 N1 P1	368.2559	1.90	367.2488	295, 81
	(d20:2)	6.0	C20 H40 O5 N1 P1	464.2782	−0.86	405.2644	292,264,128
	(d22:2)	6.5	C22 H44 O5 N1 P1	492.3097	−1.22	433.2957	375,180

**Table 3:** Identification of sphingolipids in breast tissue using UHPLC in “Organic Phase Extract”.

Class	Fatty acid	Retention Time(min)	Molecular Formula	Observed Mass	Error (ppm)	Calculated Mass	MS/MS fragments (m/z)
**Cer**	(d18:1/12:0) **(IS)**	3.0	C30 H58 O3 N1	526.4484292	−1.76	481.4495	283,270,88
	(d18:1/16:0)	3.0	C35 H68 O5 N1	582.5112221	−1.93	537.5121	311,298,88
	(d18:1/18:1)	2.9	C37 H70 O5 N1	608.5272988	−2.63	563.5277	102
	(d18:1/23:2)	2.9	C43 H80 O5 N1	690.6053293	−1.49	631.5903	390,347,235
	(d18:1/24:1)	2.9	C43 H82 O5 N1	692.6212052	−2.32	647.6216	546,390,237
	(d18:2/16:0)	3.0	C35 H66 O5 N1	580.4956225	−2.11	535.4964	256,104
	(d18:2/23:0)	2.9	C42 H80 O5 N1	678.6055060	−2.22	633.6060	102
	(d18:2/24:1)	2.9	C43 H80 O5 N1	690.6053805	−2.00	645.6060	316,168
	(d18:1/25:0) **(IS)**	2.9	C44 H86 O5 N1	708.6525276	−2.30	663.6529	495,439,102
**DHCer**	(d18:0/16:0)	3.0	C34 H70 O3 N1	584.5269029	−2.06	539.5277	280,255,237
	(d18:0/18:1)	2.9	C37 H72 O5 N1	610.5425634	−1.91	565.5434	102
	(d18:0/21:2)	2.9	C41 H78 O5 N1	664.5900060	−1.97	605.5747	618,364
**LacCer**	(d18:1/12:0) **(IS)**	8.0	C42 H80 O13 N1	806.5628915	0.26	805.5551	190,146,102
**SM**	(d18:1/21:1)	7.2	C46 H90 O8 N2 P1	829.646159	−2.36	770.6302	392,168,78
	(d18:2/22:0)	7.2	C45 H90 O6 N2 P1	785.6525117	1.64	784.6458	184,86
	(d18:1/12:0) **(IS)**	7.3	C35 H72 O6 N2 P1	647.5118463	1.78	646.5050	184,102
**DHSM**	(d18:0/18:1)	7.2	C41 H84 O6 N2 P1	731.6055663	1.82	730.5989	184,102
	(d18:0/24:2)	7.1	C47 H94 O6 N2 P1	813.6838264	1.57	812.6771	184,86
	(d18:0/22:1)	7.2	C45 H91 O6 N2 P1	787.6683	1.27	786.6615	102

IS- Internal Standard; CerP- Ceramide 1-Phosphate; So-Sphingosine/Sphinganine; S1P- Sphingosine 1-Phosphate; Cer-Ceramide, DHCer- Dihydro Ceramide; LacCer- Lactosyl Ceramide; SM-Sphingomyelin; DHSM-DihydroSphingomyelin.

### Relative abundance of sphingolipids in breast cancer

**Figure 1:**
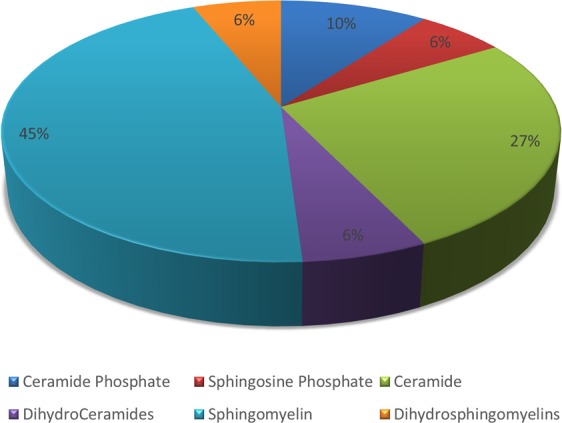
Pie chart representing the abundance of sphingolipid metabolites in breast tissue.

A total of 49 sphingolipid metabolites were observed in breast cancer patients (Fig. 1). Of all the metabolites, ceramide phosphates comprised only 10% while sphingosine phosphate constituted only 6%. A vast majority of the metabolites however belonged to ceramide and sphingomyelin groups comprising 33% and 51% of the total species, respectively.

### PLS-DA model for biomarker identification and selection

**Figure 2:**
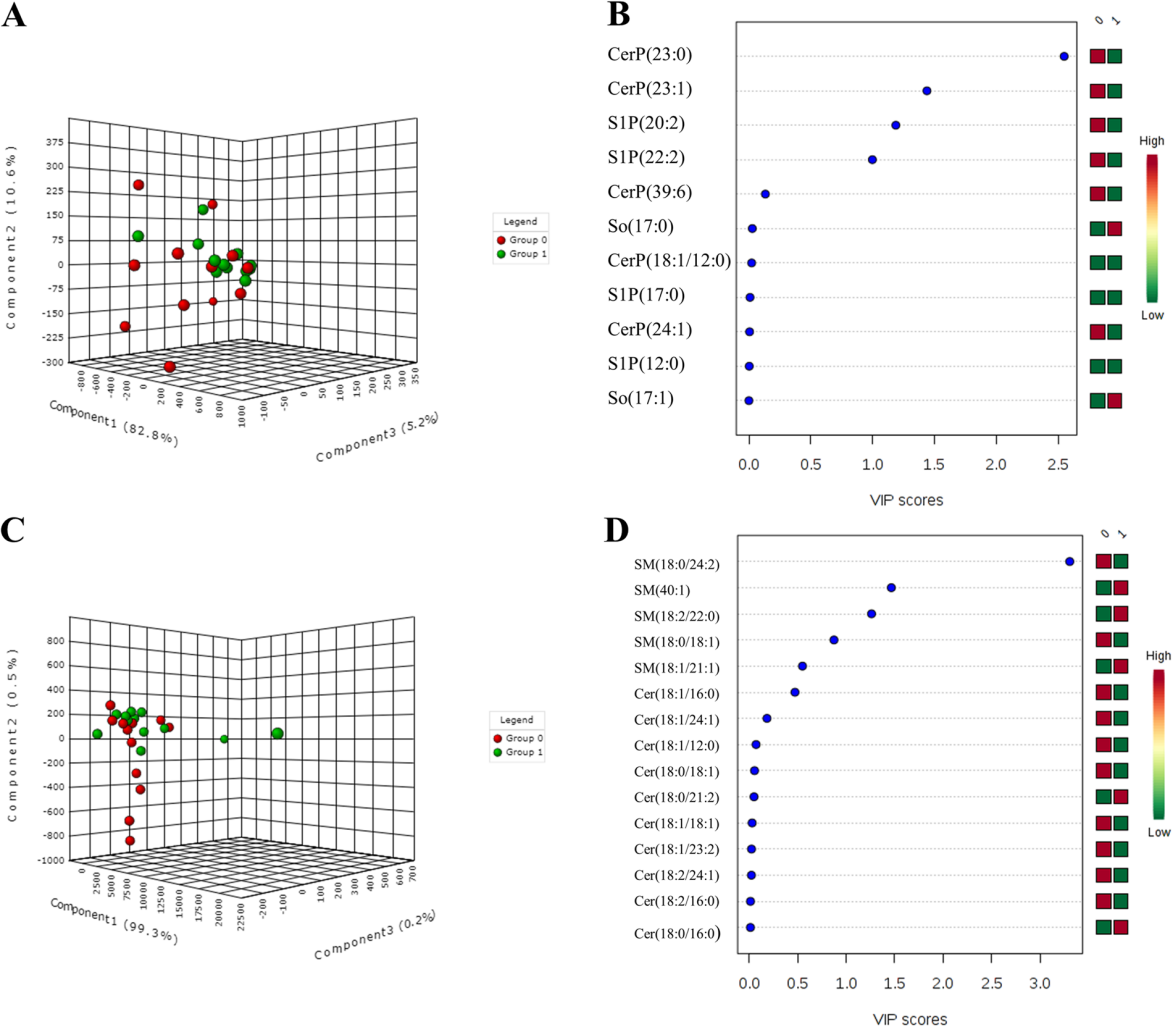
(**A**) 3D score plot (**B**) VIP score plot for sphingosine and ceramide phosphates and (**C**) 3D score plot (**D**) VIP score plot for ceramides and sphingomyelins. Group 0 (red) indicates tumor tissues and Group 1(green) indicates adjacent normal breast tissues.

Due to high-dimensionality of the lipidomics data, multivariate analysis was performed to determine sphingolipid metabolites that were significantly altered in tumor and adjacent normal tissues (Fig. 2). The variables were not well separated from each other between the two groups, as shown by 3D-score plot diagram (2A, 2C). However, Variable Importance in Projection (VIP) score values for CerP(23:0), CerP(23:1), S1P(20:2), S1P(22:2), SM(18:0/24:2), SM(18:2/22:0), SM(40:1) were found to be >1 suggesting that these metabolites can be utilized as predictivel biomarkers in breast cancer (2B, 2D).

### Dysregulation of sphingolipid levels in breast cancer

**Figure 3:**
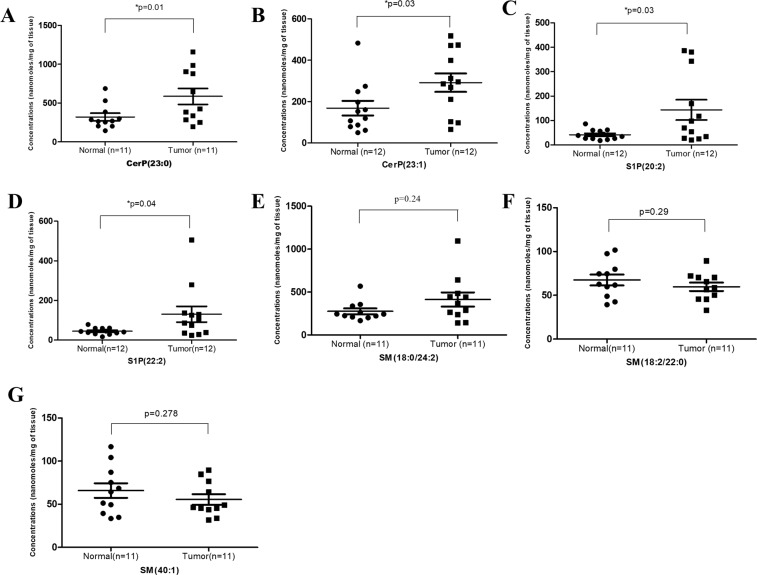
Levels of sphingolipid metabolites in breast tumor and adjacent normal tissue samples (**A**) CerP(23:0), (**B**) CerP(23:1), (**C**) S1P(20:2), (**D**) S1P(22:2), (**E**) SM(18:0/24:2), (**F**) SM(18:2/22:0), (**G**) SM(40:1).

The levels of sphingolipid metabolites having VIP score values >1 were compared in tumor and adjacent normal tissues of breast cancer patients as shown in Fig. 3. The levels of ceramide phosphates CerP(23:0) and CerP(23:1) were found to be significantly higher in tumor tissues as compared to adjacent normal tissues. Further, there was significant upregulation in the levels of sphingosine phosphates S1P(20:2) and S1P(22:2) in tumor tissues. The level of sphingomyelin SM(18:0/24:2) was also found to be high in tumor tissues, although the difference was not statistically significant. On the contrary, the level of two sphingomyelin species SM(18:0/24:2) and SM(18:2/22:0) were downregulated in tumor tissues but the difference did not reach statistical significance may be due to small sample size.

### Assessment of diagnostic potential and predictive ability of sphingolipids

#### ROC analysis for the individual sphingolipid metabolites

**Figure 4:**
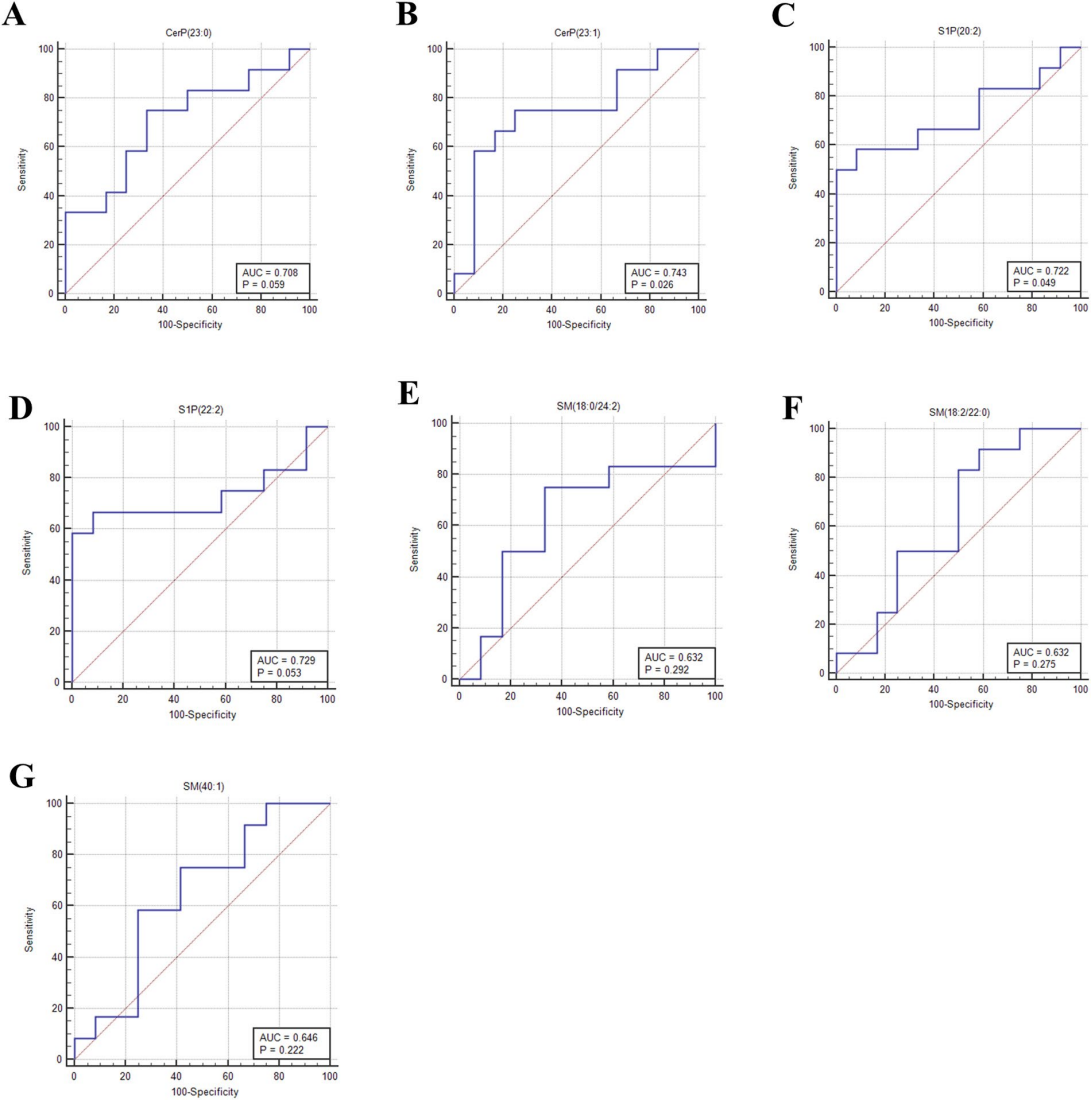
ROC curves for evaluating the diagnostic potential of sphingolipids. (**A**) CerP(23:0), (**B**) CerP(23:1), (**C**) S1P(20:2), (**D**) S1P(22:2), (**E**) SM(18:0/24:2), (**F**) SM(18:2/22:0), (**G**) SM(40:1).

ROC curve analysis was performed to determine the diagnostic potential of each sphingolipid metabolite (Fig. 4). Area under curve (AUC) was used to assess the performance of predicted biomarkers. All the above mentioned sphingolipid metabolites particularly ceramide phosphates and sphingosine phosphates were found to have a fair predictive ability in discriminating breast tumor tissues from adjacent normal tissues with AUC = 0.708 (95% CI- 0.489 to 0.874, p = 0.059) with 75% sensitivity and 66.67% specificity for CerP(23:0) (Fig. 4A), AUC = 0.743 (95% CI- 0.525 to 0.898, p = 0.026) with 75% sensitivity and 75% specificity for CerP(23:1) (Fig. 4B), AUC = 0.722 (95% CI- 0.503 to 0.884, p = 0.049) with 58.33% sensitivity and 91.67% specificity for S1P(20:2) (Fig. 4C), AUC = 0.729 (95% CI - 0.511 to 0.888, p = 0.053) with 58.33% sensitivity and 100% specificity for S1P(22:2) (Fig. 4D), AUC = 0.632 (95% CI - 0.413 to 0.817, p = 0.29) with 75% sensitivity and 66.67% specificity for SM(18:0/24:2) (Fig. 4E), AUC = 0.632 (95% CI- 0.413 to 0.817, p = 0.27) with 83.33% sensitivity and 50% specificity for SM(18:2/22:0) (Fig. 4F) and AUC = 0.646 (95% CI- 0.426 to 0.828, p = 0.22) with 58.33% sensitivity and 75% specificity for SM(40:1) (Fig. 4G).

#### ROC analysis for combination of sphingolipid metabolites

**Figure 5:**
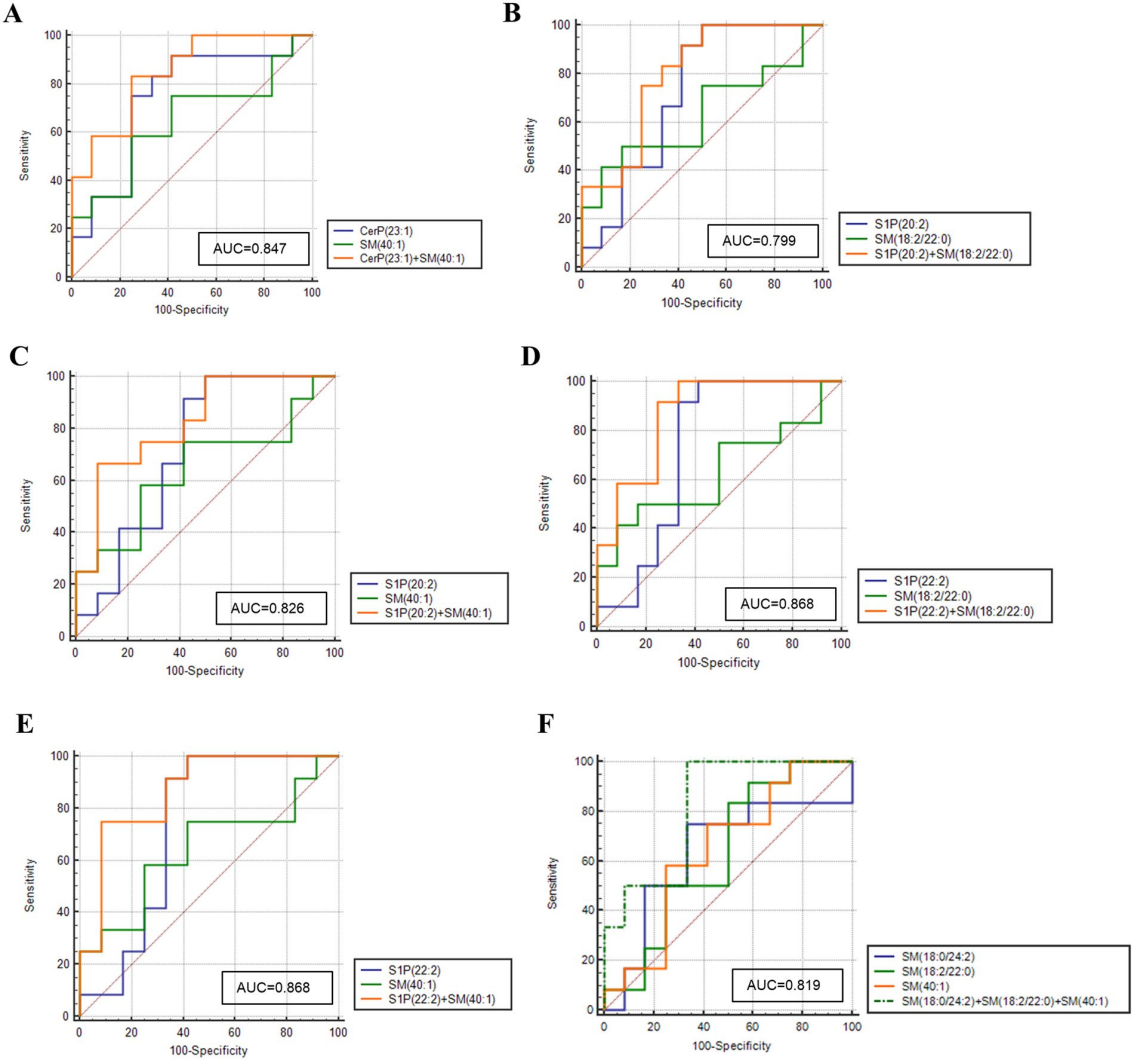
ROC curve for the combination of sphingolipid metabolites. (**A**) CerP(23:1)/SM(40:1); (**B**) S1P(20:2)/SM(18:2/22:0); (**C**) S1P(20:2)/SM(40:1); (**D**) S1P(22:2)/SM(18:2/22:0); (**E**) S1P(22:2)/SM(40:1); (**F**) SM(18:0/24:2)/SM(18:2/22:0)/SM(40:1).

Further, we evaluated the AUC scores for the combination of metabolites to determine whether the set of metabolites could be used to discriminate tumor tissues from adjacent normal breast tissues. The cumulative AUC scores were estimated for the 22 combinations of 7 sphingolipid metabolites (Supplementary Table 3). Of the various determined combinations, 6 set of metabolites were found to have improved AUC values (≥ 0.8) as compared to individual metabolites and are represented in Fig. 5.

### Association of sphingolipid levels with clinicopathological characteristics and proliferation potency in breast cancer

**Figure 6:**
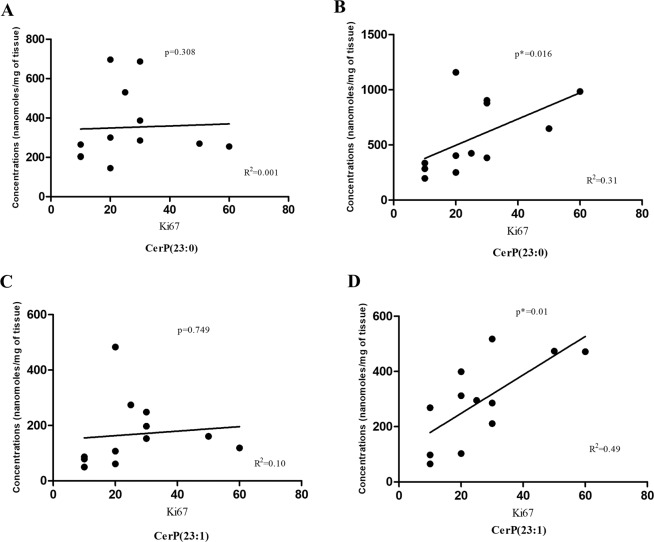
Correlation between Ki67 index and CerP(23:0) levels in (**A**) adjacent normal (**B**) tumor tissue and CerP(23:1) levels in (**C**) adjacent normal and (**D**) tumor tissues.

The correlation of aforementioned sphingolipid metabolites with age, tumor stage, tumor grade was also determined. No significant relationship was found between the clinical features and sphingolipid levels in breast cancer cohort owing to small sample size. Further, in order to determine the role of SPLs in breast cancer aggressiveness, we next investigated the association between the levels of SPLs and Ki-67 index. It was found that the Ki67 levels were positively correlated to ceramide phosphate CerP(23:0) and CerP(23:1) levels in tumor tissues (Fig. 6B, 6D). However, no correlation was found between Ki67 and ceramide phosphate levels in adjacent normal tissues (Fig. 6A, 6C). This study revealed that the ceramide phosphates might have a possible role as a biomarker in cancer proliferation and aggressiveness. No significant associations were found between Ki67 index and the level of other sphingolipid metabolites.

### Hierarchical clustering heatmap of sphingolipid metabolites in breast cancer patients

**Figure 7:**
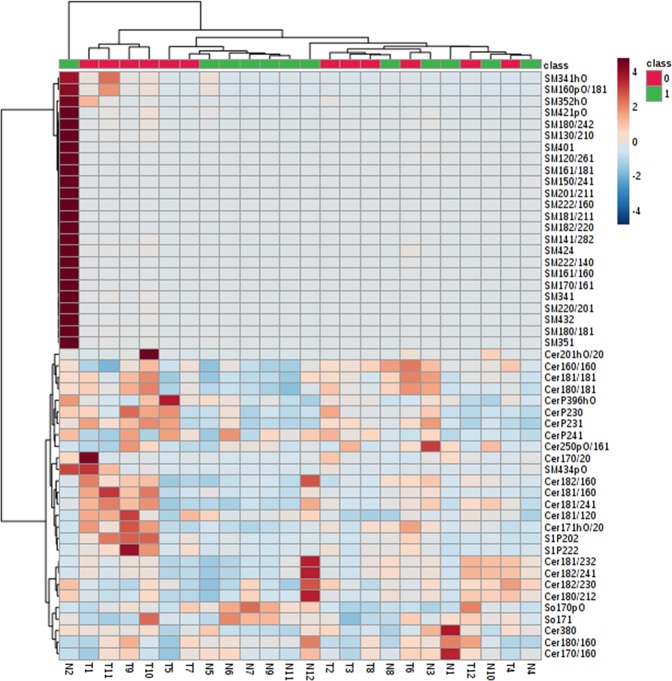
Heatmap showing levels of SPLs in breast tissues. Columns represent the adjacent normal and tumor tissues while rows represent the sphingolipid species. Shades of blue and maroon represent reduction and elevation in the levels of SPLs with respect to the median value. Group 0 (red) indicates tumor tissues and Group 1(green) indicates adjacent normal breast tissues.

Based on the levels of SPLs, a heatmap of the hierarchical clustering was plotted. The metabolites were classified into two clusters corresponding mainly to sphingomyelins and ceramides. Within each cluster, two sub clusters were observed (Fig. 7). Further, it was found that ceramide phosphates and sphingosine phosphates belonged to same sub-cluster indicating that these metabolites might have a common role in breast cancer.

### Dysregulation of sphingolipid metabolizing genes in breast cancer

**Figure 8:**
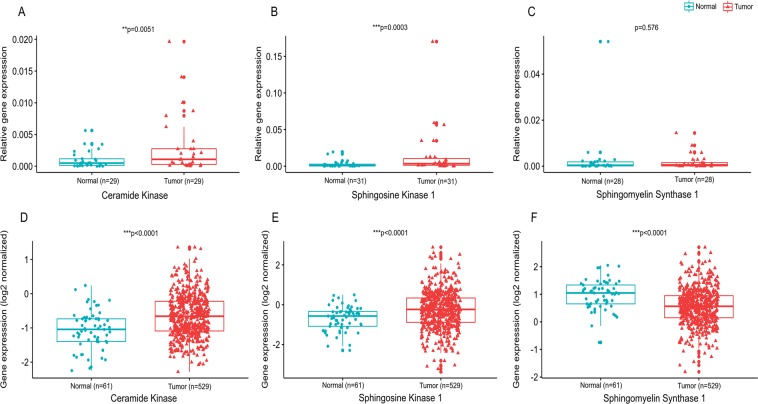
Expression of sphingolipid metabolizing genes. (**A**) Ceramide Kinase, (**B**). Sphingosine Kinase 1 and (**C**). Sphingomyelin synthase 1 in local cohort and (**D**). Ceramide Kinase, (**E**). Sphingosine Kinase 1 and (**F**). Sphingomyelin synthase 1 in TCGA cohort.

To validate alterations in the levels of sphingolipid metabolites in breast cancer patients, the expression analysis of genes involved in their synthesis was carried out in local cohort as well as The Cancer Genome Atlas (TCGA) cohort. There was a significant increase in the levels of Ceramide Kinase (CERK) gene in tumor tissues as compared to adjacent normal tissues in both local as well as TCGA cohort (Fig. 8A, 8D). The levels of Sphingosine Kinase 1 (SPHK1) were also found to be significantly upregulated in tumor tissues in both the cohorts (Fig. 8B, 8E). No significant difference was observed in the expression of Sphingomyelin Synthase 1 (SGMS 1) between tumor and adjacent normal tissues in local cohort (Fig. 8C). In contrast, TCGA cohort exhibited significant downregulation of SGMS1 in tumor tissues as compared to adjacent normal tissues (Fig. 8F). Further, ROC curve analysis was also performed for the abovementioned genes in local and TCGA cohort (Supplementary Fig. 1). The AUC values for CERK and SPHK1 depicted a fair and significant potential to discriminate between tumor and adjacent normal tissues in local (Supplementary Fig. 1A, 1B) and TCGA cohort (Supplementary Fig. 1D and 1E) respectively. A fair AUC score was also observed for SGMS1 gene and was found to be significant only in TCGA cohort (Supplementary Fig. 1F, 1C).

## Discussion

Metabolomics has recently emerged as a highly robust technique for the discovery of biomarkers to diagnose, monitor or predict the risk of disease^12^. Identification and prediction of a biomarker, however, also requires precise data analysis by using appropriate statistical measures. Unfortunately, this area is often overlooked by researchers and only few studies have utilized proper statistical approaches for the biomarker selection. The current study was aimed to address this lacuna by combining highly efficient UHPLC-High Resolution (orbitrap) Mass Spectrometry (HRMS) approach with multivariate analysis to identify the sphingolipid biomarkers of clinical significance. A comprehensive profiling of SPLs was performed in tumor and adjacent normal tissues obtained from breast cancer patients. The study enlisted all the possible sphingolipid species in these patients and led to the identification of 49 SPLs belonging to 6 sub-categories. To visualize the lipid variations among the two groups (tumor and adjacent normal tissue), partial least square discriminant analysis (PLSDA) was employed. PLSDA is a method used to sharpen the separation among different observation groups and predict the discriminant variable using the Variable Importance in Projection (VIP) scores. VIP values determine the impact of individual variable that contribute to separation among groups in the PLS-DA models. Generally, VIP values of 0.7–0.8 have been considered acceptable for the variable selection while the values equal to or greater than 1.0 are the most influential^13^. In this cohort, we report a total of seven potential SPLs including CerP(23:0), CerP(23:1), S1P(20:2), S1P(22:2), SM(18:0/24:2), SM(18:2/22:0) and SM(40:1) with VIP values>1, however we did not find any metabolites having VIP values within the range of 0.7–0.8. Similar approach using VIP score has been utilized to predict the pathological response to neo-adjuvant chemo-radiation therapy (NCRT) in locally advanced rectal cancers^14^. As far as SPLs are concerned, there is only one study that has used VIP score to identify 52 differential sphingolipid metabolites in A270 Human Ovarian Cancer Cell Line and its Taxol resistant strain^15^. Similarly, VIP values were used to predict the potential glycerophospholipid biomarkers in breast cancer cell lines MCF-7 and MDA-MB-231^16^. The satisfactory prediction performance of the above studies underlines the importance of VIP scores for the biomarker prediction in clinical studies. To the best of our knowledge, none of the studies have tried to predict sphingolipid biomarker in breast cancer patients using VIP values.

Among selected species, the levels of ceramide phosphates CerP(23:0) and CerP(23:1) were significantly increased in tumor tissues as compared to adjacent normal tissues. Ceramide 1- phosphate (Cer1P) has been reported to play important role in cancer associated pathways including cell growth, survival, proliferation^17,18^, inflammation and migration^19,20^. Cer1P is synthesized through the phosphorylation of ceramide by the enzyme Ceramide Kinase (CERK). Therefore, to confirm the above observations, we also analyzed the expression of CERK in breast tissues. The present study reports significantly high levels of CERK in tumor tissues as compared to adjacent normal tissues in local as well as TCGA cohort. CERK has also been reported earlier to promote tumor cell growth, survival and mammary tumor reccurrence^21,22^. High expression of CERK has been found to be associated with poor prognosis in breast cancer^23^. Contrary to our studies, a recent report suggested that CERK expression in TCGA cohort is downregulated in tumor tissues as compared to adjacent normal breast tissues^24^. However, the difference among the two studies in TCGA analysis can be attributed to heterogeneity of the samples and utilization of different platforms.

To establish the role of ceramide phosphates in breast cancer aggressiveness, we analyzed the association of ceramide phosphates to Ki-67 index reported in these patients. It was found that ceramide phosphate levels have a significant positive correlation to high Ki-67 index in tumor tissues. An earlier report suggested that high ceramide levels are associated with low proliferation potency in breast cancer patients^24^. Although, ceramide phosphates are formed from the ceramides, the two metabolites have antagonistic effects in cancer^25,26^. The present study is the first report on the levels of ceramide phosphates and CERK expression in breast cancer patients and clinical correlations of ceramide phosphates with Ki67 index, making it a pioneer study in this field.

A good biomarker selection further requires the identification of metabolites that are capable of discriminating between two groups (diseased vs healthy) with high sensitivity and specificity. Numerous studies have suggested ROC curve as most statistically valid method for evaluation of biomarker performance^27–29^. Therefore, to validate the significance of these metabolites as biomarkers, we further used ROC curve analysis. It was observed that ceramide phosphates CerP(d23:0) and CerP(d23:1) have a fair diagnostic potential with AUC values greater than 0.7.

Another significant finding of our study is the presence of these rare odd carbon chain ceramide phosphates CerP(23:0) and CerP(23:1) in patient samples. Although, almost all natural occurring fatty acids are even numbered, a recent study reported that odd carbon chain fatty acids also contribute <1% of human plasma composition^30^. Further, the authors reported four significantly measurable odd carbon chain fatty acids, C15:0, C17:0, C17:1^31^ and C23:0^32^. The odd carbon chain fatty acids C15:0 and C17:0 have also been acquiring attention within the scientific community as biomarkers for type II diabetes mellitus (T2D) and coronary heart disease (CHD). Studies have shown that there is a negative association between the levels of circulating odd carbon chain fatty acids and the risk for metabolic disease^33,34^. On the contrary, C15:0 and C17:0 were found to be positively associated with etiology of T2D and CHD^35,36^. The possible reasons for the presence of circulating C23:0 in rat brain was found to be either the exogenous intake through milk or endogenous production by the elongation of odd carbon chain fatty acid C17:0^37,38^. Recently, a genome-wide association meta-analysis of circulating odd carbon chain fatty acids revealed that C23:0 is predominantly a component of sphingolipids and is associated with a common variation in ceramide synthase CERS4 gene^39^. However, there is no report underlining the role of odd carbon chain SPLs in cancer patients till date.

Other sphingolipid metabolites selected according to VIP score were sphingosine phosphates S1P(20:2) and S1P(22:2). Sphingosine 1-phosphates (S1P) has been recognized as crucial regulators of sphingolipid rheostat as they reduce pro-apoptotic ceramide and augment survival signaling^40^. In the present study, tumor samples exhibited significantly higher levels of S1P as compared to adjacent normal tissues. Universally high levels of S1P in breast cancer patients have also been reported earlier however they did not observe individual species of S1P as reported in the present study^10^. Further, a value of AUC >0.7 by ROC analysis suggested the diagnostic potential of these sphingolipid metabolites in breast cancer patients. S1P is formed from the sphingosine by the action of enzyme Sphingosine Kinase (SPHK) which exists in two isoforms- SPHK1 and SPHK2. Out of the two, SPHK1 is the most common form involved in the cancer cell growth and survival^41^. In the present study, we also analyzed the expression of SPHK1 in breast cancer in local and TCGA cohort. The levels of SPHK1 in breast tumor tissues were found to be significantly higher than the adjacent normal tissues in both the cohorts. These observations are consistent with the recent study which reports increased expression of SPHK1 in biliary tract cancer in TCGA cohort^42^. High levels of SPHK1 were shown to enhance tumor formation in breast cancer MCF-7 cells^43^. Overexpression of SPHK1 enzyme has also been reported in hepatocellular carcinoma and adrenocortical carcinoma tissues^44,45^. The S1P/SPHK1 axis has also been reported earlier to promote the pancreatic cancer growth by regulating the expression of pancreatic stellate cells^46^.

Sphingolipid metabolites have also been reported to participate in the process of cellular proliferation and contribute to tumor progression^47,48^. In ovarian cancer, S1P has been recognized to promote proliferation and stimulate chemotactic migration and invasion^49,50^. However, we did not find any significant correlation between S1P and Ki67 in these patients which could be due to small sample size in the current study.

The present study further demonstrated that the levels of two sphingomyelins SM(18:2/22:0), SM (40:1) were low while one SM(18:0/24:2) were high in tumor tissues as compared to adjacent normal tissue, but the difference was not statistically significant. Sphingomyelins are the important constituents of lipid rafts along with the cholesterol and glycerophospholipids and thus involved in the regulation of numerous signaling pathways^51^. There is contradictory evidence on the levels of sphingomyelins in cancer. A reduction in the levels of sphingomyelins has been reported in colon cancer tissues^52^. Reduced plasma levels of sphingomyelins SM(16:0), SM(24:0) and SM(24:1) have also been observed in A549 and U118 cancer cell lines^53^. Contrary to our results, Nagahashi and colleagues reported high concentration of sphingomyelins in breast tumor samples^10^. To confirm our findings, we also determined the level of Sphingomyelin synthase 1(SGMS1) in breast cancer tissues. In the local cohort, we did not observe any significant difference in the mRNA expression of SGMS1 between tumor and adjacent normal tissues, though it was found to be significantly downregulated in TCGA cohort. A recent study also suggests that the expression of SGMS1 is downregulated and is associated with sphingolipid reprogramming and worse prognosis in melanoma^54^. Though ROC curve analysis (AUC >0.6) of the sphingomyelins suggested their moderate potential as biomarkers, further studies with large sample size must be conducted to understand the clinical relevance of these species.

The clinical performance of the metabolites can be improved considerably by combining multiple metabolites in a panel. In the present study, we also aimed to evaluate the AUC values for 22 combinations of 7 metabolites. A considerable increase in the AUC score for 6 combinations: CerP(23:1)/SM(40:1); S1P(20:2)/SM(18:2/22:0); S1P(20:2)/SM(40:1); S1P(22:2)/SM(18:2/22:0); S1P(22:2)/SM(40:1); SM(18:0/24:2)/SM(18:2/22:0)/SM(40:1) was observed suggesting that these sphingolipid metabolite sets can be used efficiently to distinguish tumor tissues from adjacent normal breast tissues. Cumulative ROC analysis has also been used to improve the biomarkers performance in other cancer types^55,56^.

All these findings conclusively ascertain the involvement of sphingolipid metabolites in breast cancer patients.

We would also like to mention that none of the SPL metabolites showed any association with clinicopathological characteristics in these patients. However, the small sample size in this study is a limiting factor and further investigation with large sample size may provide a concrete evidence in this regard.

To conclude, the present study reports novel sphingolipid metabolites which may have potential as biomarkers in breast cancer. A major finding is the presence of odd carbon chain ceramide phosphates which could clearly distinguish between the tumor and adjacent normal tissues with high sensitivity and specificity. Further, a high positive association with Ki67 reinforce the assumption that these odd carbon SPL species might have a predictive role in cancer proliferation and aggressiveness.

## Materials and Methods

### Chemicals and solutions

The internal standard (IS) cocktail (LM-6005) consisting of 25 µM each of nine uncommon SPLs in ethanol including Sphinganine (d17:0), Sphingosine (d17:1), Sphinganine-1-Phosphate (d17:0) and Sphingosine-1-Phosphate (d17:1), Ceramide (d18:1/12:0), Ceramide (d18:1/25:0), Ceramide-1-Phosphate (d18:1/12:0), Hexosyl Ceramide (d18:1/12:0), Lactosyl Ceramide (d18:1/12:0) and Sphingomyelin (d18:1/12:0) was purchased from Avanti Polar Lipids (Alabaster, AL). Ammonium formate (NH_4_HCO_2_), potassium hydroxide (KOH), acetic acid (CH_3_COOH) and formic acid (HCOOH) were purchased from Sigma-Aldrich (St. Louis, MO, USA). LC-MS-grade chloroform (CHCl_3_), methanol (CH_3_OH) and isopropanol (IPA) were purchased from Merck (Darmstadt, Germany). All other chemicals used were of analytical grade.

### Sample collection

The tumor and adjacent normal tissues were collected from the breast cancer patients after surgical resection from the Department of General Surgery, PGIMER. The samples were obtained after informed consent and the protocol was approved from Institutional Ethics Committee, PGIMER (IEC-12/2017–787) and Panjab University Institute Ethics Committee (PUIEC/2019/154/A/01/03). The study was conducted according to the ethical guidelines stated in Helsinki Declaration. A total of 31 cases were included in this study. Clinical details were recorded and a pathological staging was done for all the patients. Clinicopathological parameters such as age, tumor type, pathological grade and TNM staging were determined for each resected specimen. The TNM classification was done according to the *American Joint Committee* on *Cancer* (AJCC) 8^th^ edition. Breast tissue samples were also characterized according to standard pathology based on the estrogen receptor (ER), progesterone receptor (PR) and human epidermal growth factor receptor 2 (Her2). The tissue samples were stored at −80 °C for sphingolipid isolation.

### Sphingolipid extraction

**Figure 9:**
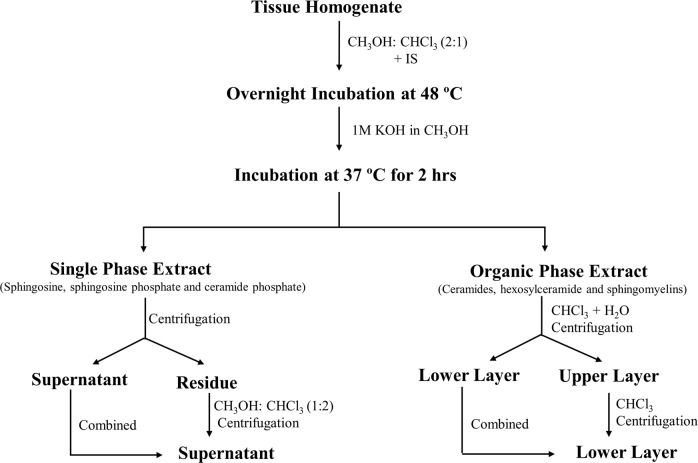
Schematic representation of the method for sphingolipid isolation from breast tissue.

The SPLs were isolated with minor modification of the method given by Shaner *et al*.^57^. The tissue was homogenized in Phosphate Buffered Saline (PBS) using a Minilys Homogenizer from Bertin Technologies (Montigny-le-Bretonneux, France) and sonorex digital ultrasonic cleaning unit (Bandelin Electronic, Berlin, Germany) at room temperature for 30 sec. After addition of 0.5 ml of CH_3_OH and 0.25 ml of CHCl_3_, 20 µl of Internal Standard (IS) was spiked into each sample. Prior to use, 1 µl (500 µg/ml) of butylated hydroxyl toluene (BHT) was added to IS for stabilization. One blank sample and one positive control were also processed along with the samples. The mixture was then incubated at 48 °C overnight to allow complete transition of SPLs. After cooling, 75 µl of 1 M KOH in CH_3_OH was added and the mixture was kept in a shaking incubator for 2 h at 37 °C to cleave glycerophospholipids. After cooling to room temperature, 5 µl of glacial acetic acid was added for the base neutralization. The mixture was divided into two halves serving as single phase extract and organic phase extract. The single-phase extract was centrifuged and the supernatant was transferred to a new vial. The residue was reextracted with 1 ml of CH_3_OH: CHCl_3_ (1:2), centrifuged and the supernatant was combined with the previous collection. To the organic phase extract, 1 ml of chloroform and 2 ml of H_2_O was added and the lower layer was collected after centrifugation. The upper phase was re-extracted with an additional 1 ml of chloroform and was combined to the lower layer residue (Fig. 9).

The single-phase extract and the organic phase extract were dried using Scanvac Centrifuge Vacumm Concentrator (Labogene, Denmark) and the residue was reconstituted in 0.2 ml of the mobile phase solvent for LC-MS/MS analysis. The mixture was then sonicated, centrifuged and the clear supernatant was transferred to auto-injector vials.

### Liquid chromatography and mass spectrometric analysis of sphingolipids

#### Single phase extract

The single-phase extract was used for analyzing the levels of sphingosine, sphingosine-1-phosphate and ceramide-1-phosphate. High resolution (Orbitrap) Mass spectrometer (HRMS) of Thermo Q-Exactive connected with Diaonex UHPLC system was used for the SPLs analysis. The chromatographic separation was performed by reverse phase LC using Column: C18 (100 mm × 1.0 mm ID; 1.7 µm particle size) and a binary solvent system with a flow rate of 0.1 ml/min. The mobile phase A1 consisted of CH_3_OH: H_2_O (60/40, v/v) with 5 mM ammonium formate and 0.3% formic acid. The mobile phase B1 consisted of CH_3_OH with 5 mM ammonium formate and 0.3% formic acid. The temperature of the C18 column was increased gradually till 40 °C to avoid carryover of Cer1P. Temperatures above 40 °C were not used in the current study as high temperature may affect the stability of the lipid species.

The A1/B1 solvent ratio was maintained at 60/40 for 1 minute followed by a gradient to 100% B1 for 5 minutes which was maintained for 1.5 minutes. The gradient was again reduced to 60/40 (A1/B1) within 0.2 minutes and was equilibrated for 2.3 minutes before the next run.

#### Organic phase extract

This phase was used for the analysis of ceramides, hexosylceramides and sphingomyelins. The separation was performed using Column:LC-NH_2_ column (100 mm × 2.1 mm ID; 1.7 µm particle size) column and a binary solvent system with a flow rate of 0.1 ml/min. The column was equilibrated prior to injection of the samples. The mobile phase A2 consisted of CH_3_CN/CH_3_OH/HCOOH (97/2/1 v/v/v) with 5 mM ammonium formate and phase B2 consisted of CH_3_OH/H_2_O/HCOOH (89/6/5) with 50 mM triethylammonium acetate. The A2/B2 gradient was maintained as follow: 100% A2 for 1 minute and was continued for 3 minutes followed by a linear gradient of 100% B2 for 1 minute. The flow was maintained for 3 minutes and then restored to 1-minute linear gradient of 100% A2 and was continued for 1 minute before the next run.

Eluted lipids were analyzed using a HRMS viz; Q-Exactive mass spectrometer (Thermo Electron, Bremen, Germany). The Xcalibur 2.2 interface was used to monitor data-dependent acquisition of lipid ions. This included a full MS scan covering 250 to 1250 Da range of mass-to-charge ratio (m/z) with a resolution of 1,40,000 and a tandem mass spectrometry (MS/MS) step (normalized collision energy: 30%; resolution: 35000). The MS/MS step was reiterated for the 5 major ions detected during the full MS scan. Dynamic exclusion time was set to 45 s.

### RNA extraction and cDNA synthesis

Total RNA was extracted from the tissue samples using PureLink RNA Mini Kit (Thermo Scientific) . The quality of the isolated RNA was checked using agarose gel electrophoresis and the yield was assessed using NanoDrop™ 2000c Spectrophotometer (Thermo Fisher Scientific, Waltham, Massachusetts, United States. Following DNase-I treatment, first strand cDNA was synthesized from 1 μg of RNA according to the protocol provided by Revert Aid First Strand cDNA Synthesis Kit (Thermo Scientific). The resulting cDNA was stored at −80 °C and was used as a substrate for qPCR.

### Quantitative polymerase chain reaction (qPCR)

The expression of the candidate genes was determined by qPCR analysis. The sequence of the primers used is given in the Table 4. The reactions were performed in duplicates in Applied Biosystems^TM^ Quant Studio^TM^ 3 Real Time PCR System (Waltham, MA,USA) using Powerup^TM^ SYBR green mastermix. The quantitative data was normalized using Glyceraldehyde 3-phosphate dehydrogenase (GAPDH) and the relative gene expression was assessed using 2^-ΔΔCt^ method.

**Table 4:** Primer sequence of the genes.

Gene Name	Forward Primer	Reverse Primer
GAPDH	5′-ACCCACTCCTCCACCTTT-3′	5′-CTGTTGCTGTAGCCAAATTCGT -3′
SPHK1	5′-AGGCTGAAATCTCCTTCACGC-3′	5′-GTCTCCAGACATGACCACCAG-3′
CERK	5′-TGGTTGGGTCTTGCCAGATAC-3′	5′-ACTTCCCACAGACGACTTGC-3′
SGMS1	5′-GTCGGAGAGCGCGATTGG3′	5′-ATGCTGTCGTCACGTTGCAC-3′

### Data analysis

**Figure 10:**
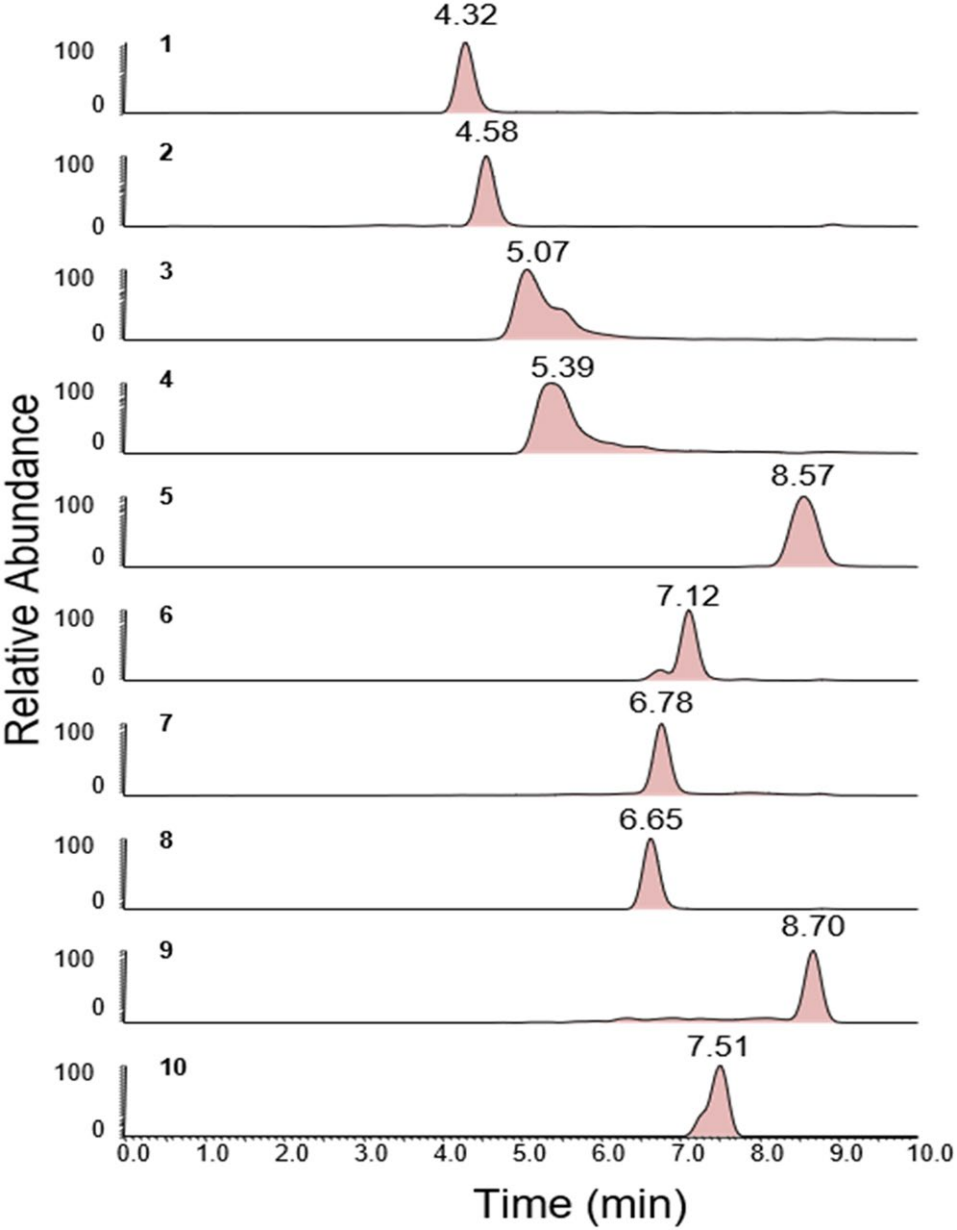
Chromatograms for the Internal Standards. **1:** C17 Sphingosine**; 2:** C17 Sphinganine; **3:** C17 Sphingosine-1- Phosphate; **4:** C17 Sphinganine-1-Phosphate; **5:** C12 Sphingomyelin; **6:** C12 Ceramide; **7:** C12 Glucosyl(β) Ceramide; **8:** C12 Lactosyl(β) Ceramide; **9:** C12 Ceramide-1-Phosphate; **10:** C25 Ceramide.

Lipid search software 4.1.30 was employed for data processing and identification of SPLs based on their accurate MS/MS data. Relative quantification was done under MS mode and peak areas of the highly-resolved extracted ion chromatograms (EICs) were integrated to obtain the area under curve of each SPL. The chromatogram for the internal standards is shown in Fig. 10.

The quantitative results were obtained by internal standard (IS) normalization and calculated as follows: Concentration of target SPL (nmol/mg of tissue) = (Area of target SPL/Area of corresponding IS) spiked concentration of IS.

All quantitative data was converted to Microsoft Excel and imported into MetaboAnalyst 4.0^58^ software for Partial Least Square-Discriminant Analysis (PLS-DA). Multivariate analysis was used to differentiate the levels of SPLs between tumor and adjacent normal specimens. Variable Importance in Projection (VIP >1) values were used for the identification of sphingolipid metabolites that can be used as potential biomarkers. VIP score >1 were considered as significant. The statistical analysis was performed with Graph Pad Prism-5 Version (Inc., La Jolla, CA, USA) and MedCalc software for Windows, version 15.0 (Ostend, Belgium). The differentially expressed SPLs and the corresponding metabolizing genes were evaluated between tumor and adjacent normal tissues using Wilcoxon t-test (non-parametric) or paired t-test (parametric) after the outlier exclusion. Unpaired t–test was used to compare the tumoral sphingolipid levels in different clinical subgroups. ROC curve was used to determine the diagnostic potential of the sphingolipid metabolites and the corresponding genes. Further, combined ROC estimation was done using logistic regression analysis. The obtained predicted probabilities were plotted as ROC curve along with the individual metabolites. Correlation of SPLs with clinicopathological characteristics was carried out using Pearson’s or Spearman’s coefficient.

### Analysis of gene expression in TCGA

The gene expression data pertaining to Breast invasive carcinoma (TCGA abbreviation BRCA) was retrieved from the *Firehose* portal of *Broad Institute (gdac.broadinstitute.org);* this dataset comprise lowess-normalized level-3 microarray data (Agilent 244 K [G4502A]) from 529 tumor and 61 adjacent normal tissues. Mann-Whitney U test (non-parametric) was used to calculate the difference in gene expression between tumor and normal tissues in TCGA cohort. All statistical analysis and image rendering was performed in R 3.4.4 statistical environment (www.r-project.org).

## Supporting Information

Supporting Information.
