# Supplementary material for: LC-HRMS based approach to identify novel sphingolipid biomarkers in breast cancer patients
Source: Sci Rep. 2020 Mar 13;10:4668. doi: 10.1038/s41598-020-61283-w (PMC7070000; doi:10.1038/s41598-020-61283-w)
Supplement: Supplementary file 1 — Supporting Information. [file 41598_2020_61283_MOESM1_ESM.docx]

**LC-HRMS based approach to identify novel sphingolipid biomarkers in breast cancer patients**

Priyanka Bhadwal^1^**,** Divya Dahiya^2^, Dhananjay Shinde^3^, Kim Vaiphei^4^, Raviswamy G H Math^5^, Vinay Randhawa^1^, Navneet Agnihotri^1*^

**Affiliations:**

1. Department of Biochemistry, Panjab University, Chandigarh, India
2. Department of General Surgery, PGIMER, Chandigarh, India
3. Department of Pathology and Microbiology, University of Nebraska Medical Centre, Omaha, USA
4. Department of Histopathology, PGIMER, Chandigarh, India
5. Mass Spectrometry Facility, NCBS/InStem, Bangalore, India

*Corresponding Author. E-mail: agnihotri.navneet@gmail.com

**Supplementary Table 1.** The fragmentation pattern of sphingolipids in “Single Phase Extract”

| **Class** | **FattyAcid** | **Retention Time(min)** | **Molecular Formula** | **Observed Mass** | **Error (ppm)** | **Calculated Mass** | **MS/MS fragments (m/z)** |
| --- | --- | --- | --- | --- | --- | --- | --- |
| **CerP** | (d39:6+hO) | 3.4 | C39 H68 O7 N1 P1 | 658.4658 | -8.96 | 693.4733 | 295,244 |
|  |  |  |  |  |  |  |  |
| **S1P** | (d12:0+pO) | 4.3 | C12 H28 O6 N1 P1 | 314.1747 | -4.77 | 313.1654 | 259, 203 |

CerP- Ceramide 1- Phosphate; S1P- Sphingosine 1-Phosphate

**Supplementary Table 2.** The fragmentation pattern of sphingolipids in “Organic Phase Extract”

| \| \| **Class** \| **Fatty acid** \| **Retention Time(min)** \| **Molecular Formula** \| **Observed Mass** \| **Error (ppm)** \| **Calculated Mass** \| **MS/MS fragments (m/z)** \| \| --- \| --- \| --- \| --- \| --- \| --- \| --- \| --- \| \| **Cer** \| (d17:1+hO/2:0) \| 5.9 \| C19 H37 O4 N1 \| 342.2656 \| -3.21 \| 343.2723 \| 257,166 \| \| (d20:1+hO/2:0) \| 3.8 \| C22 H43 O4 N1 \| 384.3127 \| -3.38 \| 385.3192 \| 102 \| \| (d16:0/16:0) \| 3.0 \| C32 H65 O3 N1 \| 570.5114 \| -1.75 \| 511.4964 \| 241,102 \| \| (d17:0/16:0) \| 3.3 \| C33 H67 O3 N1 \| 526.519 \| 1.71 \| 525.5121 \| 283,270,88 \| \| (d38:0) \| 1.4 \| C38 H77 O3 N1 \| 596.5969 \| 2.01 \| 595.5903 \| 102 \| \| (d25:0+pO/16:1) \| 2.5 \| C41 H81 O4 N1 \| 650.6106 \| -2.77 \| 651.6166 \| 102 \| \|  \|  \|  \|  \|  \|  \|  \|  \| \| **SM** \| (d16:1/16:0) \| 7.3 \| C37 H75 O6 N2 P1 \| 675.5432 \| 1.33 \| 674.5363 \| 184, 102 \| \| (d17:0/16:1) \| 7.3 \| C38 H77 O6 N2 P1 \| 689.5591 \| 0.87 \| 688.5519 \| 236,221,184, 102 \| \| (d13:0/21:0) \| 7.1 \| C39 H81 O6 N2 P1 \| 705.5902 \| 1.13 \| 704.5832 \| 248,61 \| \| (d12:0/22:1) \| 7.2 \| C39 H79 O6 N2 P1 \| 703.5743 \| 1.56 \| 702.5676 \| 184 \| \| (d34:1+hO) \| 7.5 \| C39 H79 O7 N2 P1 \| 763.5622 \| -2.49 \| 718.5625 \| 102 \| \| (d16:0+pO/18:1) \| 7.5 \| C39 H79 O7 N2 P1 \| 719.5693 \| 1.39 \| 718.5625 \| 168 \| \| (d16:1/18:1) \| 7.2 \| C39 H77 O6 N2 P1 \| 701.5592 \| 0.71 \| 700.5519 \| 277,184, 102 \| \| (d35:1) \| 7.2 \| C40 H81 O6 N2 P1 \| 717.5889 \| 2.93 \| 716.5832 \| 315 \| \| (d35:2+hO) \| 6.9 \| C40 H79 O7 N2 P1 \| 731.5696 \| 0.96 \| 730.5625 \| 184, 102 \| \| (d22:2/14:0) \| 7.2 \| C41 H81 O6 N2 P1 \| 729.5902 \| 1.10 \| 728.5832 \| 312,102 \| \| (d12:0/26:1) \| 7.2 \| C43 H87 O6 N2 P1 \| 759.6363 \| 2.24 \| 758.6302 \| 184, 102 \| \| (d22:2/16:0) \| 7.2 \| C43 H85 O6 N2 P1 \| 757.6213 \| 1.32 \| 756.6145 \| 112,68 \| \| (d15:0/24:1) \| 7.1 \| C44 H89 O6 N2 P1 \| 773.6527 \| 1.16 \| 772.6458 \| 398 \| \| (d20:1/21:1) \| 7.1 \| C46 H91 O6 N2 P1 \| 799.6683 \| 1.25 \| 798.6615 \| 184 \| \| (d22:0/20:1) \| 7.1 \| C47 H95 O6 N2 P1 \| 815.7000 \| 0.74 \| 814.6928 \| 283,255,218 \| \| (d42:1+pO) \| 7.4 \| C47 H95 O7 N2 P1 \| 831.6950 \| 0.60 \| 830.6877 \| 102 \| \| (d14:1/28:2) \| 7.1 \| C47 H91 O6 N2 P1 \| 811.6682 \| 1.36 \| 810.6615 \| 184 \| \| (d42:4) \| 7.1 \| C47 H89 O6 N2 P1 \| 809.6519 \| 2.10 \| 808.6458 \| 146 \| \| (d43:2) \| 7.1 \| C48 H95 O6 N2 P1 \| 827.6989 \| 2.05 \| 826.6928 \| 102 \| \| (d43:4+pO) \| 6.8 \| C48 H91 O7 N2 P1 \| 839.6632 \| 1.19 \| 838.6564 \| 291,247,133 \| \| \| --- \| --- \| --- \| --- \| --- \| --- \| --- \| --- \| --- \| --- \| --- \| --- \| --- \| --- \| --- \| --- \| --- \| --- \| --- \| --- \| --- \| --- \| --- \| --- \| --- \| --- \| --- \| --- \| --- \| --- \| --- \| --- \| --- \| --- \| --- \| --- \| --- \| --- \| --- \| --- \| --- \| --- \| --- \| --- \| --- \| --- \| --- \| --- \| --- \| --- \| --- \| --- \| --- \| --- \| --- \| --- \| --- \| --- \| --- \| --- \| --- \| --- \| --- \| --- \| --- \| --- \| --- \| --- \| --- \| --- \| --- \| --- \| --- \| --- \| --- \| --- \| --- \| --- \| --- \| --- \| --- \| --- \| --- \| --- \| --- \| --- \| --- \| --- \| --- \| --- \| --- \| --- \| --- \| --- \| --- \| --- \| --- \| --- \| --- \| --- \| --- \| --- \| --- \| --- \| --- \| --- \| --- \| --- \| --- \| --- \| --- \| --- \| --- \| --- \| --- \| --- \| --- \| --- \| --- \| --- \| --- \| --- \| --- \| --- \| --- \| --- \| --- \| --- \| --- \| --- \| --- \| --- \| --- \| --- \| --- \| --- \| --- \| --- \| --- \| --- \| --- \| --- \| --- \| --- \| --- \| --- \| --- \| --- \| --- \| --- \| --- \| --- \| --- \| --- \| --- \| --- \| --- \| --- \| --- \| --- \| --- \| --- \| --- \| --- \| --- \| --- \| --- \| --- \| --- \| --- \| --- \| --- \| --- \| --- \| --- \| --- \| --- \| --- \| --- \| --- \| --- \| --- \| --- \| --- \| --- \| --- \| --- \| --- \| --- \| --- \| --- \| --- \| --- \| --- \| --- \| --- \| --- \| --- \| --- \| --- \| --- \| \| Cer-Ceramide, SM-Sphingomyelin  **Supplementary Table 3.**  ROC analysis for combination of sphingolipid metabolites.   \|  \| **AUC Values** \| \| \| \| \| \| \| \| \| --- \| --- \| --- \| --- \| --- \| --- \| --- \| --- \| --- \| \| **Combination** \| **A** \| **B** \| **C** \| **D** \| **E** \| **F** \| **G** \| **Cumulative** \| \| **A+B** \| .708 \| .743 \|  \|  \|  \|  \|  \| .736 \| \| **A+C** \| .708 \|  \| .722 \|  \|  \|  \|  \| .778 \| \| **A+D** \| .708 \|  \|  \| .729 \|  \|  \|  \| .778 \| \| **A+E** \| .708 \|  \|  \|  \| .632 \|  \|  \| .778 \| \| **A+F** \| .708 \|  \|  \|  \|  \| .632 \|  \| .729 \| \| **A+G** \| .708 \|  \|  \|  \|  \|  \| .646 \| .778 \| \| **B+C** \|  \| .743 \| .722 \|  \|  \|  \|  \| .764 \| \| **B+D** \|  \| .743 \|  \| .729 \|  \|  \|  \| .778 \| \| **B+E** \|  \| .743 \|  \|  \| .632 \|  \|  \| .778 \| \| **B+F** \|  \| .743 \|  \|  \|  \| .632 \|  \| .785 \| \| **B+G** \|  \| .743 \|  \|  \|  \|  \| .646 \| **.847** \| \| **C+D** \|  \|  \| .722 \| .729 \|  \|  \|  \| .729 \| \| **C+E** \|  \|  \| .722 \|  \| .632 \|  \|  \| .771 \| \| **C+F** \|  \|  \| .722 \|  \|  \| .632 \|  \| **.799** \| \| **C+G** \|  \|  \| .722 \|  \|  \|  \| .646 \| **.826** \| \| **D+E** \|  \|  \|  \| .729 \| .632 \|  \|  \| .764 \| \| **D+F** \|  \|  \|  \| .729 \|  \| .632 \|  \| **.868** \| \| **D+G** \|  \|  \|  \| .729 \|  \|  \| .646 \| **.868** \| \| **E+F** \|  \|  \|  \|  \| .632 \| .632 \|  \| .736 \| \| **E+G** \|  \|  \|  \|  \| .632 \|  \| .646 \| .771 \| \| **F+G** \|  \|  \|  \|  \|  \| .632 \| .646 \| .646 \| \| **E+F+G** \|  \|  \|  \|  \| .632 \| .632 \| .646 \| **.819** \| \| |  |  |  |  |  |  |  |  |  |  |  |  |
| --- | --- | --- | --- | --- | --- | --- | --- | --- | --- | --- | --- | --- | --- | --- | --- | --- | --- | --- | --- | --- | --- | --- | --- | --- | --- | --- | --- | --- | --- | --- | --- | --- | --- | --- | --- | --- | --- | --- | --- | --- | --- | --- | --- | --- | --- | --- | --- | --- | --- | --- | --- | --- | --- | --- | --- | --- | --- | --- | --- | --- | --- | --- | --- | --- | --- | --- | --- | --- | --- | --- | --- | --- | --- | --- | --- | --- | --- | --- | --- | --- | --- | --- | --- | --- | --- | --- | --- | --- | --- | --- | --- | --- | --- | --- | --- | --- | --- | --- | --- | --- | --- | --- | --- | --- | --- | --- | --- | --- | --- | --- | --- | --- | --- | --- | --- | --- | --- | --- | --- | --- | --- | --- | --- | --- | --- | --- | --- | --- | --- | --- | --- | --- | --- | --- | --- | --- | --- | --- | --- | --- | --- | --- | --- | --- | --- | --- | --- | --- | --- | --- | --- | --- | --- | --- | --- | --- | --- | --- | --- | --- | --- | --- | --- | --- | --- | --- | --- | --- | --- | --- | --- | --- | --- | --- | --- | --- | --- | --- | --- | --- | --- | --- | --- | --- | --- | --- | --- | --- | --- | --- | --- | --- | --- | --- | --- | --- | --- | --- | --- | --- | --- | --- | --- | --- | --- | --- | --- | --- | --- | --- | --- | --- | --- | --- | --- | --- | --- | --- | --- | --- | --- | --- | --- | --- | --- | --- | --- | --- | --- | --- | --- | --- | --- | --- | --- | --- | --- | --- | --- | --- | --- | --- | --- | --- | --- | --- | --- | --- | --- | --- | --- | --- | --- | --- | --- | --- | --- | --- | --- | --- | --- | --- | --- | --- | --- | --- | --- | --- | --- | --- | --- | --- | --- | --- | --- | --- | --- | --- | --- | --- | --- | --- | --- | --- | --- | --- | --- | --- | --- | --- | --- | --- | --- | --- | --- | --- | --- | --- | --- | --- | --- | --- | --- | --- | --- | --- | --- | --- | --- | --- | --- | --- | --- | --- | --- | --- | --- | --- | --- | --- | --- | --- | --- | --- | --- | --- | --- | --- | --- | --- | --- | --- | --- | --- | --- | --- | --- | --- | --- | --- | --- | --- | --- | --- | --- | --- | --- | --- | --- | --- | --- | --- | --- | --- | --- | --- | --- | --- | --- | --- | --- | --- | --- | --- | --- | --- | --- | --- | --- | --- | --- | --- | --- | --- | --- | --- | --- | --- | --- | --- | --- | --- | --- | --- | --- | --- | --- | --- | --- | --- | --- | --- | --- | --- | --- | --- | --- | --- | --- | --- | --- | --- | --- | --- | --- | --- | --- | --- | --- | --- | --- | --- | --- | --- | --- | --- | --- | --- | --- | --- | --- | --- | --- | --- | --- | --- | --- | --- | --- | --- |

1. CerP(23:0) B. CerP (23:1) C. S1P(20:2), D. S1P(22:2), E. SM(18:0/24:2), F. SM(18:2/22:0), G. SM(40:1).


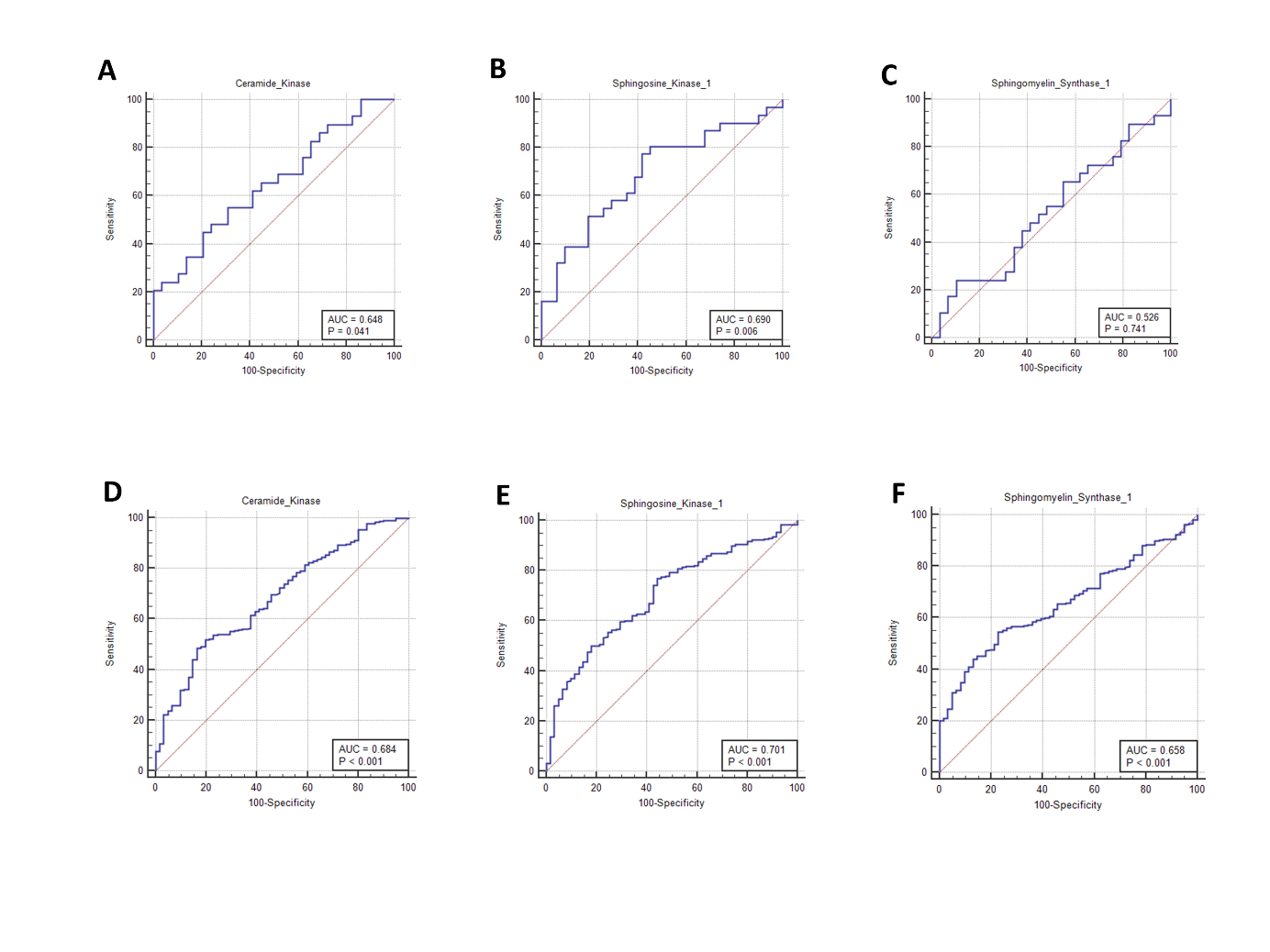


**Supplementary Fig 1.** ROC curves for sphingolipid metabolizing genes A. Ceramide Kinase, B. Sphingosine Kinase 1 and C. Sphingomyelin synthase 1 in local cohort and D. Ceramide Kinase, E. Sphingosine Kinase 1 and F. Sphingomyelin synthase 1 in TCGA cohort.
